# Height and nigral neuron density in Parkinson’s disease

**DOI:** 10.1186/s12883-022-02775-2

**Published:** 2022-07-11

**Authors:** Laura Saari, Emmilotta A. Backman, Pia Wahlsten, Maria Gardberg, Valtteri Kaasinen

**Affiliations:** 1 Clinical Neurosciences, Faculty of Medicine, University of Turku, Turku, Finland; 2 Neurocenter, Turku University Hospital, Turku, Finland; 3 Forensic Medicine Unit, Finnish Institute for Health and Welfare, Southwest Finland region, Helsinki, Finland; 4 Department of Pathology, Turku University Hospital and Institute of Biomedicine, University of Turku, Turku, Finland

**Keywords:** Parkinson’s disease, Parkinsonism, Height, Substantia nigra, Pathology

## Abstract

**Background:**

The dopaminergic system modulates growth hormone secretion and previous results have suggested a link between short stature and an increased risk of Parkinson’s disease (PD).

**Methods:**

In 36 Lewy body spectrum disease (LBD) cases (PD = 22) and 19 controls, nigral TH-positive neuron densities were measured postmortem from midbrain sections and corrected with the Abercrombie method. Body measurements were collected from autopsies or patient records. Our aim was to investigate the possible relationship between height and the density of neurons in the substantia nigra pars compacta (SNc).

**Results:**

SNc neuron density (n/mm^2^) had an inverse association with height, (R^2^ = 0.317, *p* < 0.0001) in patients. The association was not explained by weight, age, sex, brain weight, medication, or disease motor severity. The association was also separately observed in patients with PD (*n* = 22), but not in subjects who died without diagnosed neurological diseases.

**Conclusions:**

Individual adult height may be connected to nigral neuron numbers in patients with LBDs, including PD.

## Introduction

Human adult height is determined by genetic factors, nutrition, socioeconomic status, age, and health, but data on the association between height and morbidity are heterogeneous. The strongest negative associations between height and mortality have been reported in respiratory and cardiovascular diseases, whereas an opposite relationship has been reported between height and the risk for cancer and musculoskeletal diseases [1]. A combined epidemiological and genetic evaluation of the possible association between height and diseases confirmed a link between height and the risk for 32 diseases but found no link with psychiatric and neurological diseases [1]. However, the mean height of individuals with Parkinson’s disease (PD) has been reported to be lower than that of controls, although the difference was significant only in men [2]. The authors hypothesized that the lower stature of PD patients may be associated with dopaminergic connections with the hypothalamic-pituitary axis during early brain development [2]. A similar association has been seen with dementia risk [3]. Studies have also associated PD with higher risk of osteoporosis, and it has been hypothesized that PD may affect into skeletal homeostasis [4]. Stature decline in PD could thus also be caused by various postural deformities in PD, such as camptocormia, antecollis (dropped head) and scoliosis [5].

The loss of dopaminergic neurons in the Substantia nigra pars compacta (SNc) is a key neuropathological change in Lewy body diseases (LBDs), but the factors that influence the number of dopaminergic neurons in PD are unclear. There is a large variation in the number of nigral neurons in patients with PD, and symptom severity, disease duration or treatment factors only partially explain the interindividual differences in SNc. Therefore, given the previously reported association between height and PD, we aimed to investigate the relationship between dopaminergic neuron density and height postmortem in patients with PD and in individuals who died without diagnosed neurological diseases.

## Methods

### Subjects and clinical data

Thirty-six postmortem cases of patients with LBDs and 19 healthy controls were included in this study. The included patients were collected from 168 postmortem cases with neuropathologically confirmed Lewy body pathology (database previously described in [6]). The inclusion criteria were available height and weight information and autopsy data, and acceptable quality of nigral tissue samples for neuronal counting. The most common clinical diagnosis was PD (*n* = 22). Retrospectively the patients were categorized to three clinical phenotypes: parkinsonism predominant (*n* = 13), dementia (*n* = 11), and mixed (*n *= 12). Height and weight measurements were collected from autopsies (*n* = 28) conducted at Turku University Hospital, where the weight was measured with a floor scale and height with a measuring stick from heels to the top of the head. For eight cases, the measurements were not available and were retrieved from clinical records (median (range) time from recorded body measurements to death was 4.0 (0.9–10.2) years). Nineteen controls (individuals who died without known neurological diseases) were individuals who were examined by forensic pathologists at the Finnish Institute for Health and Welfare, as described [7].

### Neuropathology

Database selection criteria and neuropathological methods have been described in detail previously [6]. Briefly, formalin-fixed and paraffin-embedded midbrain at the level of origin of the third cranial nerve was sectioned at 8 µm thick sections and stained with Luxol Fast Blue to evaluate borders of substantia nigra pars compacta (magnification 1.0) and with tyrosine hydroxylase (TH) immunohistochemistry for calculation of dopaminergic neurons. The sections were scanned with a Pannoramic P25 Flash slide scanner and analyzed with CaseViewer software version 2.3.0.99276 (3D HISTECH Ltd, Budapest, Hungary). TH-positive neurons were systematically manually calculated from the SNc (magnification 20.0). The cell count was corrected with the Abercrombie method using the diameter of TH-positive nuclei (magnification 63.0 or higher) as described [6]. The median (range) time from death to autopsy was 5 (2–8) days, and the median time from autopsy to neuropathological examination was 23 (12–73) days (missing data for 2 patients). Controls were analyzed using the same protocol. The median (range) time from death to autopsy was 3 (1–8), and the median time from autopsy to neuropathological examination was 20 (11–56).

### Statistics

SPSS Statistics 25 for Windows (IBM Corp, Armonk, NY, USA) was used for statistical analyses. Correlations between neuron density and clinical factors were evaluated with Pearson’s correlation or Spearman’s correlation as appropriate. Linear regression analysis by the enter method was performed within the correlating variables. Differences between groups were analysed with independent sample t-tests or chi-square tests as appropriate. Only two-tailed tests were evaluated and *p*-values < 0.05 were considered significant. The normality of distribution was evaluated with histograms and the Shapiro–Wilk test.

## Results

**Fig. 1:**
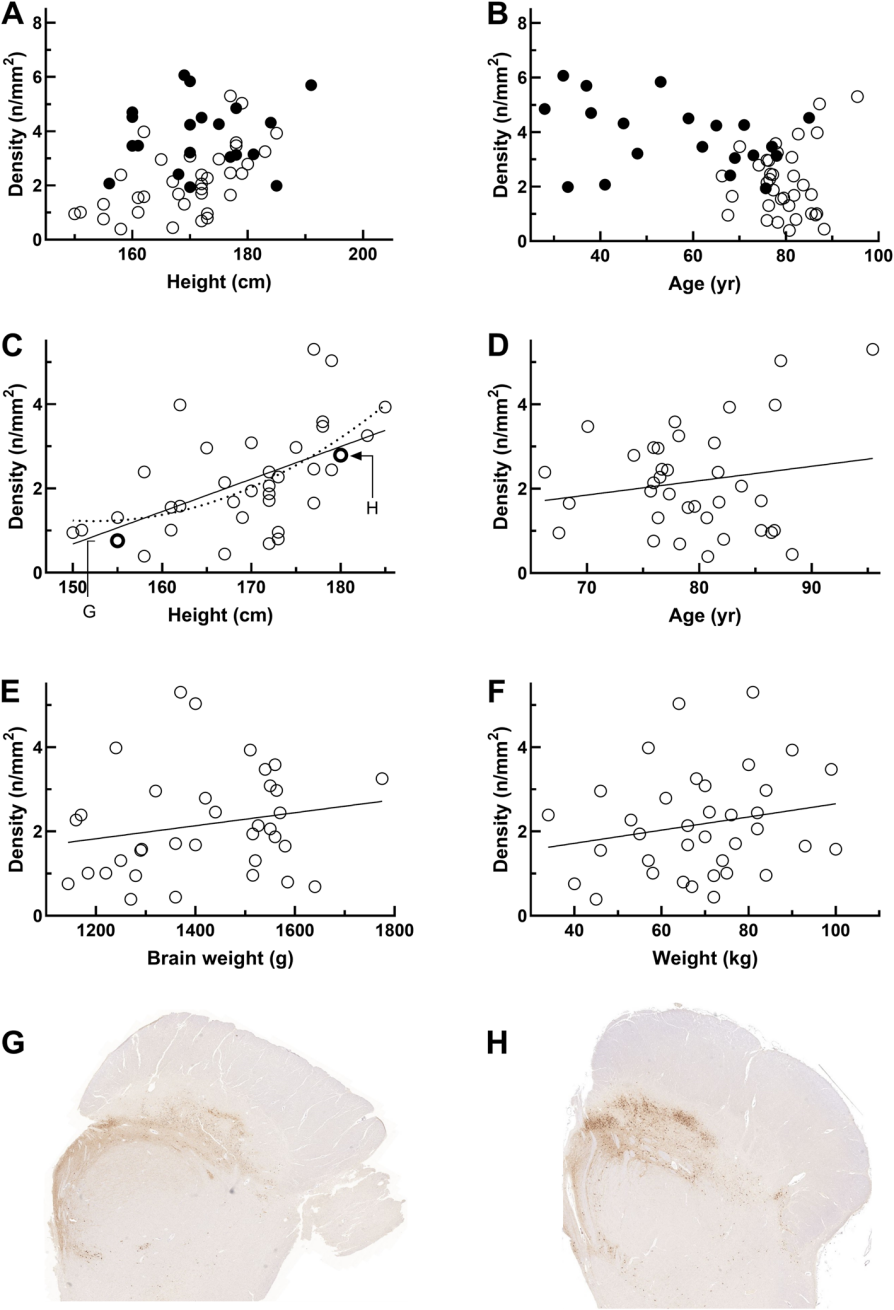
Substantia nigra pars compacta dopaminergic neuron density in relation to height, weight, age and brain weight. There were no sex differences in patient SNc neuron density over the whole sample (*p* = 0.25). **A** Height in patients with LBDs (open circles) and healthy controls (solid circles) in relation to neuron density (*n* = 55, *r* = 0.402, *p* = 0.002). **B** Age in relation to neuron density in patients with LBDs (open circles) and healthy controls (solid circles) (nonsignificant). **C** Linear (*r* = 0.563, *p* < 0.0001) and quadratic (*r* = 0.592, *p* = 0.001) relationships between height and neuron density in patients with LBDs (*n* = 36). Two individual patients are pointed out and correspond to nigral sections in Panels G and H. **D** Nonsignificant correlation between age and neuron density in patients. **E** Nonsignificant correlation between brain weight and neuron density in patients. **F** Nonsignificant correlation between weight and neuron density in patients. **G** A representative section of TH staining in the SNc of a PD patient with short stature (height 155 cm, female, age at death = 76 years, HY stage 5, symptom duration 18 years). Note the faint staining intensity indicating fewer neuromelanin containing dopaminergic neurons compared to the nigral section in Panel **H**. **H** A representative section of TH staining in the SNc of a PD patient with tall stature (height 180 cm, male, age at death = 74 years, HY stage 5, symptom duration 14 years). Note the stronger staining intensity compared to Panel **G**

The demographic and clinical characteristics of the subjects are presented in Table 1. A simple linear regression was calculated to predict SNc neuron density based on height. A significant regression equation was found (*n* = 36, F(1, 34) = 15.790, *p* < 0.0001, moderate R^2^ = 0.317). The predicted SNc neuron density was equal to -10.873 + 0.077 (SNc neuron density) n/mm^2^ (Fig. 1C). Representative sections of TH staining in the SNc of a PD patient with short and tall stature are presented in Fig. 1G and H. The association was similarly significant in patients with clinical PD (*n* = 22, F(1, 18) = 11.093, *p* = 0.004, R^2^ = 0.381, predicted neuron density equal to -12.389 + 0.085 (SNc neuron density) n/mm^2^). In the patient sample (*n* = 36), there were no significant correlations between SNc neuron density and weight (*r* = 0.20, *p* = 0.24, Fig. 1F), brain weight (*r* = 0.20, *p* = 0.26, Fig. 1E), age (*r* = 0.17, *p* = 0.32, Fig. 1D), Hoehn and Yahr stage (*r* = 0.08, *p* = 0.69), levodopa equivalent daily dose of dopaminergic medication (LEDD) (*r* = 0.07, *p* = 0.70), interval between symptom onset to death (*r* = 0.10, *p* = 0.63), interval between diagnosis to death (*r* = 0.08, *p* = 0.68), delay between death and autopsy (*r* = -0.08, *p* = 0.67) or delay between autopsy to neuropathological examination (*r* = -0.09, *p* = 0.62). The regression equation was slightly stronger when stature declines were included in the model (*n* = 36, F(1, 34) = 16.760, *p* < 0.0001, R^2^ = 0.330). The stature decline itself did not correlate with the neuron density (*r* = -0.15, *p* = 0.392) when it was evaluated mathematically, k = K/(1 + (- i))^n^, where k is height at the age of 50, K is the final height, n is the years lived after 50 and i is an annual decline of height percentage in decimal (for females 0.13% and males 0.09%) [8].

**Table 1:** Demographic and clinical characteristics of the studied subjects

	**Controls**	**All Patients**	**PD patients**
*n*	19	36	22
Sex, m/f	11/8	23/13	14/8
Age at death, years	55.9 (18) [19]	79.4 (6.2) [36]	79.1 (5.8) [22]
Height, cm	171.8 (9.7) [19]	169.3 (9.0) [36]	169.0 (9.6) [22]
Weight, kg	84.9 (18) [19]	68.9 (15.9) [36]	66.2 (15.9) [22]
Brain weight, g	-	1433 (153) [35]	1420 (169) [22]
Parkinsonism onset to death, years	-	8.4 (5.1) [25]	9.2 (5.0) [20]
Diagnosis to death, years	-	4.3 [6] [29]	6.6 [7] [20]
LEDD^1^, mg	-	455 (372) [32]	520 (334) [21]
Hoehn and Yahr stage	-	4.1 (0.83) [26]	4.1 (0.85) [19]
SNc area, mm^2^	30.6 (5.9) [19]	31.9 (7.3) [36]	31.9 (7.2) [22]
SNc neuron count, n	121 (43.6) [19]	69.2 (41.5) [36]	63.2 (38.0) [22]
SNc neuron density, n/mm^2^	3.94 (1.21) [19]	2.17 (1.23) [36]	1.99 (1.17) [22]

Values are means (SD) [n] or medians [interquartile range, IQR] [n]. ^1^ Levodopa equivalent daily dose of dopaminergic medication

When excluding the eight patients with body measurements from clinical records, the correlation with height remained significant (Pearson *r* = 0.584, *p* = 0.001). There were no sex differences in patient SNc neuron density (*p* = 0.25). The correlation between height and SNc neuron density also remained significant when patients with depression were excluded from the analysis and when sex and age were included as covariates in the linear regression model (*r* = 0.58, *p* = 0.004, β = 0.082, SD = 0.023). Quadratic correlation (r^2^ = 0.351) demonstrated the association slightly better than linear correlation (r^2^ = 0.317) (Fig. 1A).

In controls, there were no significant correlations between SNc neuron density and height (*r* = 0.115, *p* = 0.64), weight (*r* = -0.143, *p* = 0.56) or age (*r* = -0.27, *p* = 0.27), and no sex difference in SNc neuron density (*p* = 0.99). In the total sample (patients and controls combined, *n* = 55), SNc neuron density correlated with height (*r* = 0.402, *p* = 0.002, Fig. 1A), weight (*r* = 0.298, *p* = 0.027), and age (*r* = -0.364, *p* = 0.006, Fig. 1B).

## Discussion

The results of the present study suggest a link between adult height and SNc neuron density in patients with LBDs. This association was seen in patients with PD, but not in controls. Short stature in LBD patients may be one of the factors that explains interindividual differences in nigral neuron numbers.

A previous study reported that a shorter young adult height increases the risk of PD decades later. The increase in risk was seen particularly in young males with short stature [2]. Our results further demonstrate that PD patients with low height at autopsy have lower numbers of dopaminergic neurons than taller patients, an effect that was not explained by differences in demographics or clinical characteristics of the disease. Although the two studies differ considerably in terms of methodology and perspective (premorbid risk increase vs. postmortem neuron counts), both studies point to a possible link between low height and greater risk for dopaminergic degeneration. Dopamine seems to modulate body size in *C. elegans* [9], and dopamine D2 receptors regulate body size through growth hormone secretion [10]. Importantly, 6-hydroxydopamine administration to rats at the perinatal stage not only causes a severe loss of striatal dopaminergic innervation but reduces the growth rate of the animals [11]. Progressed PD often leads to inadequate nutritional status, weight loss and immobility, which can cause depleted bone density [4]. Moreover, in mice 1-methyl-4-phenyl-1,2,3,6-tetrahydropyridine seems to cause bone loss, but treatment with levodopa further reduces bone mass suggesting specific dopamine dependent mechanism [12]. Though our results should be considered preliminary and need verification with larger samples, they support a theory of shared factors determining adult height and vulnerability to neuronal degeneration.

We observed the association between SNc neuronal density and height only in patients with Lewy body pathology, and quadratic correlation demonstrated a slightly stronger relationship than the linear correlation. We did not see a similar correlation in somewhat younger individuals who had died without known neurological diseases and showed no neuropathological signs of degenerative diseases. It is possible that the association between height and SNc neuron density is specific to vulnerable neuronal populations in patients who develop LBDs. It should also be noted that correlation coefficients between neuronal density and height were moderate and SNc neuron density is just one of many factors affecting adult height in patients with Lewy body spectrum diseases. It is also possible that SNc neurons do not directly reflect hypothalamic dopamine function as a decline in SNc may not be associated with a decline of dopaminergic neurons in the arcuate nucleus. The hypothalamic neurodegeneration has in fact been associated with the symptom severity of PD, endocrine and autonomic dysfunction and some non-motor symptoms of PD but not with the severity of SNc degeneration [13]. Nevertheless, further studies with larger samples of LBD patients and neurologically healthy individuals are needed, including hypothalamic sampling besides SNc. It is also of interest that even though weight loss and low BMI have been associated with PD severity [14], we were not able to find a relationship between BMI and SNc pathology. It is clear that multiple factors affect BMI in advanced PD, such as gastrointestinal symptoms, dysphagia and anhedonia associated with depression [15]. It is likely that these factors could overrun and mask the possible minor association between BMI and neuronal loss particularly in relatively small sample of patients. The timing of the body measurements may also obscure possible associations to neurodegeneration. Most measurements were performed at autopsy, and weight measurements postmortem do not necessarily reflect the weight during adult life due to weight loss at end stages of the disease before death. The error caused by the age-related stature decline [8] was nevertheless corrected in our model. A limitation of this study is the retrospective collection of clinical data from patient histories which led to some unavailable and missing data. Potential factors such as the age of symptom onset, genetic background and highest adult height should be considered for further studies. As a clinicopathological investigation, the present study is also limited by delays between clinical and neuropathological findings.

To summarize, our results point to a link between adult height and dopaminergic neuronal density in patients with PD. This finding is in line with a previous study focusing on the risk of PD and height. This could reflect the interaction between dopaminergic neurotransmission and growth hormone secretion in human development.

## Data Availability

The datasets generated and/or analysed during the current study are not publicly available due information security issues of individual health data but pseudonymized data are available from the corresponding author on reasonable request.

## Abbreviations

BMI: Body Mass Index
LBD: Lewy Body Diseases
PD: Parkinson’s Disease
SNc: Substantia Nigra Pars Compacta
TH: Tyrosine Hydroxylase
